# Explaining disparities in colorectal cancer screening among five Asian ethnic groups: A population-based study in California

**DOI:** 10.1186/1471-2407-10-214

**Published:** 2010-05-19

**Authors:** Annette E Maxwell, Catherine M Crespi, Cynthia M Antonio, Peiyun Lu

**Affiliations:** 1 Division of Cancer Prevention & Control Research, School of Public Health/Jonsson Comprehensive Cancer Center, University of California, Los Angeles, USA; 2 Department of Biostatistics, School of Public Health, University of California, Los Angeles, USA

## Abstract

**Background:**

Data from the California Health Interview Survey (CHIS) indicate that levels and temporal trends in colorectal cancer (CRC) screening prevalence vary among Asian American groups; however, the reasons for these differences have not been fully investigated.

**Methods:**

Using CHIS 2001, 2003 and 2005 data, we conducted hierarchical regression analyses progressively controlling for demographic characteristics, English proficiency and access to care in an attempt to identify factors explaining differences in screening prevalence and trends among Chinese, Filipino, Vietnamese, Korean and Japanese Americans (N = 4,188).

**Results:**

After controlling for differences in gender and age, all Asian subgroups had significantly lower odds of having ever received screening in 2001 than the reference group of Japanese Americans. In addition, Korean Americans were the only subgroup that had a statistically significant decline in screening prevalence from 2001 to 2005 compared to the trend among Japanese Americans. After controlling for differences in education, marital status, employment status and federal poverty level, Korean Americans were the only group that had significantly lower screening prevalence than Japanese Americans in 2001, and their trend to 2005 remained significantly depressed. After controlling for differences in English proficiency and access to care, screening prevalences in 2001 were no longer significantly different among the Asian subgroups, but the trend among Korean Americans from 2001 to 2005 remained significantly depressed. Korean and Vietnamese Americans were less likely than other groups to report a recent doctor recommendation for screening and more likely to cite a lack of health problems as a reason for not obtaining screening.

**Conclusions:**

Differences in CRC screening trends among Asian ethnic groups are not entirely explained by differences in demographic characteristics, English proficiency and access to care. A better understanding of mutable factors such as rates of doctor recommendation and health beliefs will be crucial for designing culturally appropriate interventions to promote CRC screening.

## Background

Colorectal cancer (CRC) is the second leading cause of cancer deaths in the United States and among the most common cancers in all racial/ethnic subgroups, including Asian Americans. Overall, the incidence and mortality of CRC is lower in Asian Americans than in Non-Hispanic whites [1]. Only few reports on the burden of CRC disaggregate Asian American populations: California Cancer Registry data indicate that the incidence of CRC is higher in Japanese American males and females than in Non-Hispanic whites and other racial/ethnic groups and Japanese American males also have a significantly higher CRC mortality [2]. Using data from the Surveillance, Epidemiology, and End Results program, Lin and colleagues demonstrated that Filipino American males are significantly less likely than other racial/ethnic groups to be diagnosed at an early stage. In addition, their 5 year survival rate is significantly lower than those of other racial/ethnic groups. Chinese American females have poorer 5-year survival after CRC than Japanese or Filipina [3]. Population-based survey data indicate that the proportion of the population over age 50 screened for CRC significantly increased in the United States and in California between 2001 and 2005 [4,5]. However, these gains did not accrue equally across all racial and ethnic groups. Using California Health Interview Survey (CHIS) data, we recently reported the prevalence of CRC screening (ever screened) in 2001, 2003 and 2005 in California among whites, blacks, Latinos, Asians and the population overall, and among five Asian American groups with adequate sample sizes for analysis, Chinese, Filipino, Japanese, Korean and Vietnamese Americans [5]. The prevalence of screening among Asians in aggregate was consistently 11 percentage points lower than among the overall population in all three years (58%, 59% and 62% in 2001, 2003 and 2005 versus 69%, 70% and 73%), and 16 percentage points lower than among whites (74%, 75% and 78%). However, analysis of the Asian subgroups revealed more nuanced differences. Among the subgroups, Japanese Americans had the highest prevalence, with age- and gender-standardized levels of 71%, 72% and 77% in 2001, 2003 and 2005, respectively, while Korean Americans had the lowest, with levels of 49%, 43% and 33%; the other groups were at intermediate levels. The decline among Korean Americans contrasted with rising trends for Chinese, Filipino, Japanese and Vietnamese Americans. Overall, the gap between the highest and lowest prevalence groups, Japanese and Korean Americans, increased during this relatively short time period. These findings highlight the importance of disaggregating Asian American subgroups when monitoring health indicators to avoid masking differences among them [6,7].

Prior studies investigating differences in cancer screening utilization among Asian American subgroups have attributed these differences to underlying variations in demographic characteristics such as education and income, English proficiency and access to health care [6-10]. The purpose of this investigation was to examine the role of these factors in explaining the observed differences in CRC screening prevalence among the five Asian American ethnic subgroups, and in particular the low levels and declining prevalence of CRC screening among Korean Americans. To this end, we conducted hierarchical regression analyses progressively controlling for demographic characteristics, English proficiency and access to care, as well as subgroup comparisons on reasons for non-adherence to screening guidelines and rates of doctor recommendation of CRC screening to identify differences in these factors which might explain screening prevalence disparities.

## Methods

The California Health Interview Survey (CHIS) is a population-based random digit dial telephone survey conducted biennially since 2001 [11]. The CHIS is designed to provide cross-sectional, population-based statewide estimates of health indicators for all major racial and ethnic groups as well as several Asian ethnic subgroups. The CHIS obtains oral informed consent from each respondent prior to conducting the survey. This analysis was exempt from Institutional Review Board (IRB) review by the University of California, Los Angeles IRB because it was based on existing data and information was recorded in such a manner that subjects could not be identified. Data analysis was conducted in 2009. We merged data from the 2001, 2003, and 2005 surveys and created an analysis dataset consisting of adults 50 years of age and older with no history of CRC who self-identified as Chinese (N = 1,432), Filipino (N = 753), Vietnamese (N = 709), Korean (N = 675) or Japanese American (N = 619). Since Asian Americans make up 12.5% of the population in California, which is a much larger proportion than the national average of 4.5% (http://quickfacts.census.gov/qfd/states/06000.html, accessed 12/15/2009), and since CHIS oversampled several Asian American groups, this data set is well suited to examining disparities in CRC screening among Asian American ethnic groups.

### Outcome variable

Our primary outcome variable was "ever received CRC screening," a binary variable. Respondents with an affirmative response to the question "Have you ever done a blood stool test, using a home test kit?" and/or an affirmative response to the question "Have you ever had a sigmoidoscopy or colonoscopy?" (in 2001 and 2003, this question also included proctoscopy) were classified as ever received CRC screening. Respondents who responded negatively to both questions were classified as never received CRC screening. Since the reason for screening was not assessed in all three years, we could not restrict the analysis to routine screening; hence results pertain to CRC screening for any reason. We used "ever received CRC screening" rather than "screened according to the guidelines" as the outcome because type of exam and time frame were not assessed in the same manner in all three years. In addition, using "ever received CRC screening" allowed us to obtain larger numbers than "screened according to the guidelines," which had small numbers in some racial/ethnic groups, and is less subject to faulty recall of timing of tests.

### Statistical analysis

Prior to conducting multivariate analyses, we examined the bivariate relationship of past CRC screening with each of our potential explanatory variables. For each Asian American group, we estimated the proportion with past CRC screening by year (2001, 2003, 2005), age (50-64 yr, ≥ 65 yr), gender, education (≤ high school, > high school), marital status (yes, no), employment status (yes, no), percent federal poverty level (0-99%, 100-199%, 200-299%, ≥ 300%), English proficiency, health insurance (yes, no), usual source of care (yes, no) and most recent doctor's visit (≤ 1 year, > 1 year). In the CHIS, English proficiency ("How well do you speak English?") was asked of non-U.S. born respondents only; we dichotomized the responses as inapplicable because U.S. born/very well versus well/not well/not at all well. Proportions were estimated using the proportion procedure for survey data (svy proportion) in Stata Version 9.1 with CHIS-provided survey sampling weights, so that the estimates are applicable to the California population of the five Asian ethnic groups.

Multivariate models for the outcome variable "ever received CRC screening" were fit using the logistic regression procedure for survey data (svy logistic) in Stata Version 9.1 with CHIS-provided survey sampling weights. Guided by the Andersen Behavioral Model of Health Services Use [12], which suggests that people's use of health services is determined by predisposing characteristics (e.g., demographics), enabling resources (e.g., language proficiency and access to health care) and health beliefs, we added the variables in this stepped approach to understand the impact of predisposing characteristics (demographics) and enabling resources (e.g., language proficiency and access to health care) on CRC screening and to test if screening disparities are fully explained by these factors. We fit four models, a base model (Model 1) estimating trends in screening prevalence among the five Asian ethnic groups adjusted for age and gender, and three models that added covariates in blocks, sequentially adjusting for demographic characteristics (Model 2), English proficiency (Model 3), and access to health care (Model 4), with each model retaining the variables in the previous model. Model 1 included as covariates Asian ethnic subgroup (Chinese, Filipino, Vietnamese, Korean, Japanese), year of survey (2001, 2003, 2005), interactions between subgroup and year, age and gender. Model 2 added education, marital status, employment status, and percent federal poverty level. Model 3 added English proficiency. Model 4 added access to health care as measured by health insurance, usual source of care and most recent doctor's visit. The joint significance of each block of added covariates was assessed using adjusted Wald tests. Health beliefs were not assessed by the CHIS in a manner that would permit inclusion in the multivariate models. However, we did perform a separate analysis of cited reasons for non-adherence to CRC screening (see below).

Japanese Americans, who had the highest screening prevalence, and the year 2001 were used as reference categories for ethnic subgroup and year, respectively. For the main effect of subgroup, the odds ratios compare each subgroup to Japanese Americans in 2001. For the main effect of year, the odds ratios compare 2003 to 2001 and 2005 to 2001 for Japanese Americans. The subgroup-by-year interaction terms allow for trends that differ from Japanese Americans in the other subgroups. The odds ratios for the interactions of subgroup with 2003 (or 2005) are ratios of odds ratios that compare the trend in each subgroup from 2001 to 2003 (or 2005) to the trend in Japanese Americans from 2001 to 2003 (or 2005). For these interaction terms, an odds ratio less than unity indicates a negative trend, while an odds ratio greater than unity indicates a positive trend compared to Japanese Americans.

We obtained a nonparametric estimate of the relationship between the log odds of ever receiving screening and age using a lowess curve, in which each smoothed value is obtained as a weighted quadratic least squares regression over a span of values of the y-axis variable, using the Stata lowess command. This curve showed that the log odds of ever receiving CRC screening increased with age until about 73 years of age and then declined (results not shown). To model this relationship, we included a quadratic term for age in the models and centered this variable.

### Reasons for not receiving CRC screening

In 2001 and 2005, respondents whose most recent endoscopy was more than 10 years ago or who had never had one were asked "What is the one most important reason why you (never had/not had) one of these exams (in the past 10 years)?" Respondents whose most recent FOBT was more than one year ago or who had never had one were asked "What is the most important reason you have (never had/not had) a home blood stool test (in the past 12 months)?" We grouped reasons for not receiving CRC screening into four categories: unaware of test, have no health problems, fear of pain/embarrassed (endoscopy only) and other reasons, then cross-classified the responses by Asian subgroup. We tested for differences in the distribution of reasons by ethnicity using chi-square tests of the null hypothesis of homogeneity, and identified cells with unusually high or low numbers under the null hypothesis using the criterion of a standardized residual with absolute value greater than 3 [13]. Data from 2001 and 2005 were combined for this analysis.

### Doctor recommendation

In 2001 and 2005, respondents whose most recent endoscopy was more than 10 years ago or who had never had one were asked "During the past 12 months, has a doctor recommended that you have a sigmoidoscopy or colonoscopy?" Respondents whose most recent FOBT was more than one year ago or who had never had one were asked "In the past 12 months, has a doctor recommended that you have a home blood stool test?" Based on these questions, we created a binary variable "Doctor recommended endoscopy and/or FOBT in the past 12 months." For each of the five Asian subgroups, we computed the proportion who had received a doctor's recommendation in 2001 and 2005, respectively. We tested for differences among the Asian subgroups within each year and tested for change over time within each subgroup using two-sample tests for differences of proportions.

The CHIS questions regarding reasons for not receiving screening and doctor recommendation were only applicable to the subset of respondents who were non-adherent to CRC screening, and the number of relevant respondents in some subgroups (e.g., Japanese Americans) was relatively small. For these reasons, we conducted unweighted analyses of these data, since proper use of the survey weights was not feasible.

## Results

Table 1 presents the estimated proportions of Asian Americans with past CRC screening by year, demographic characteristics, English proficiency and access to health care measures. Estimates by survey year show that Japanese Americans had the highest prevalence of past screening while Korean Americans had the lowest, and the proportion screened among Korean American declined from 2001 to 2005. These patterns are consistent with previously reported age- and gender-standardized estimates [5]. Other estimates suggest differences in screening prevalence associated with age, gender, education, marital status, employment status, income, English proficiency and health care access, motivating adjustment for these variables in the multivariate analysis [6-10].

**Table 1:** Estimated proportions of Asian Americans with past CRC screening by sample characteristics, with 95% confidence intervals

	Asian American Group				
	**Japanese (N = 619)**	**Chinese (N = 1,432)**	**Filipino (N = 753)**	**Korean (N = 675)**	**Vietnamese (N = 709)**

Year					
2001	.74 (.66, .81)	.59 (.54, .65)	.56 (.48, .63)	.49 (.42, .57)	.51 (.45, .57)
2003	.74 (.66, .82)	.63 (.57, .69)	.54 (.45, .63)	.40 (.31, .50)	.58 (.49, .67)
2005	.81 (.76, .86)	.64 (.58, .69)	.65 (.57, .72)	.34 (.56, .42)	.60 (.52, .68)

Age					
50-64 yr	.64 (.57, .71)	.54 (.49, .58)	.54 (.48, .59)	.38 (.31, .44)	.52 (.46, .58)
≥ 65 yr	.84 (.79, .89)	.74 (.70, .78)	.65 (.57, .72)	.47 (.39, .54)	.70 (.63, .76)

Gender					
Male	.74 (.68, .81)	.61 (.56, .66)	.61 (.53, .68)	.37 (.31, .44)	.53 (.46, .60)
Female	.77 (.72, .83)	.63 (.59, .68)	.57 (.51, .62)	.43 (.37, .49)	.61 (.53, .68)

Education					
≤ 12 yrs	.79 (.72, .87)	.59 (.54, .64)	.58 (.49, .67)	.38 (.30, .45)	.60 (.55, .66)
>12 yrs	.75 (.69, .80)	.65 (.61, .70)	.58 (.53, .64)	.44 (.37, .50)	.48 (.39, .57)

Marital status					
Not married	.69 (.60, .77)	.66 (.60, .72)	.55 (.46, .63)	.40 (.31, .48)	.60 (.51, .69)
Married	.81 (.76, .86)	.61 (.58, .65)	.60 (.54, .65)	.41 (.35, .47)	.56 (.50, .62)

Employment status					
Not employed	.84 (.80, .88)	.69 (.65, .73)	.60 (.53, .68)	.47 (.41, .53)	.67 (.61, .72)
Employed	.62 (.54, .70)	.54 (.48, .59)	.57 (.50, .63)	.34 (.28, .41)	.41 (.33, .49)

Federal poverty level					
0-99% FPL	.75 (.49, 1.00)	.59 (.52, .66)	.46 (.30, .63)	.42 (.32, .52)	.58 (.51, .65)
100-199% FPL	.73 (.60, .86)	.64 (.57, .70)	.55 (.45, .65)	.39 (.30, .49)	.61 (.51, .70)
200-299% FPL	.80 (.71, .89)	.54 (.45, .64)	.58 (.46, .69)	.38 (.26, .51)	.51 (.36, .67)
≥ 300% FPL	.77 (.71, .82)	.66 (.60, .71)	.62 (.56, .68)	.42 (.33, .51)	.54 (.42, .65)

English proficiency					
NA*/very well	.80 (.76, .85)	.71 (.64, .77)	.62 (.56, .68)	.54 (.34, .74)	.56 (.31, .81)
Well/not well/not at all well	.62 (.52, .73)	.60 (.57, .64)	.54 (.47, .60)	.40 (.35, .45)	.57 (.52, .62)

Health insurance					
No	.32 (.00, .69)	.27 (.18, .35)	.26 (.12, .40)	.27 (.18, .36)	.58 (.46, .69)
Yes	.77 (.73, .81)	.67 (.63, .70)	.60 (.56, .65)	.46 (.41, .52)	.57 (.52, .62)

Usual source of care					
No	.31 (.14, .49)	.26 (.18, .35)	.30 (.12, .47)	.27 (.18, .37)	.23 (.05, .41)
Yes	.78 (.74, .82)	.66 (.62, .69)	.59 (.55, .64)	.44 (.38, .49)	.59 (.54, .64)

Most recent doctor's visit					
>1 yr ago	.41 (.24, .58)	.23 (.16, .31)	.45 (.26, .64)	.24 (.14, .34)	.30 (.16, .45)
Past yr	.80 (.76, .84)	.68 (.65, .72)	.59 (.54, .64)	.45 (.34, .50)	.60 (.55, .65)

* This question was not asked if respondent was born in the US.

Results obtained using survey proportion estimation with the CHIS-provided survey weights.

The results of the multivariate analyses, which progressively control for differences in demographic characteristics, English proficiency and access to care among the five Asian ethnic groups, are presented in Table 2. Joint significance tests indicated that each block of added covariates was statistically significant. All models control for gender and age. Model 1, which estimates differences in screening among ethnic subgroups after controlling for gender and age, revealed that compared to the reference group of Japanese Americans, all other Asian subgroups had significantly lower odds of having ever received screening in 2001. Odds ratios ranged from 0.41 among Korean Americans to 0.62 among Chinese Americans. Ethnicity-by-year interactions indicated that Korean Americans were the only subgroup that had a statistically significant decline in screening prevalence from 2001 to 2005 compared to the trend among Japanese Americans.

**Table 2:** Multivariate logistic models for characteristics associated with ever receiving colorectal cancer screening among Chinese, Filipino, Vietnamese, Korean and Japanese Americans

	Model 1			Model 2			Model 3			Model 4		
	**OR**	**95% CI**	**p**	**OR**	**95% CI**	**p**	**OR**	**95% CI**	**p**	**OR**	**95% CI**	**p**

Ethnicity												
Japanese (ref)												
Chinese	0.62	(0.39, 1.00)	**.049**	0.73	(0.46, 1.17)	.188	0.96	(0.59, 1.56)	.862	1.03	(0.62, 1.70)	.921
Filipino	0.53	(0.31, 0.91)	**.022**	0.60	(0.35, 1.02)	.060	0.67	(0.39, 1.17)	.159	0.61	(0.35, 1.06)	.082
Korean	0.41	(0.24, 0.71)	**.001**	0.49	(0.29, 0.83)	**.008**	0.67	(0.38, 1.17)	.159	0.83	(0.45, 1.54)	.559
Vietnamese	0.46	(0.28, 0.77)	**.003**	0.67	(0.40, 1.14)	.138	0.90	(0.51, 1.56)	.702	0.80	(0.46, 1.41)	.442

Year												
2001 (ref)												
2003	1.05	(0.58, 1.92)	.867	1.01	(0.56, 1.82)	.974	1.02	(0.57, 1.82)	.958	0.98	(0.55, 1.76)	.948
2005	1.59	(0.91, 2.79)	.102	1.61	(0.94, 2.78)	.085	1.64	(0.94, 2.86)	.082	1.74	(1.00, 3.05)	.052

Ethnicity × Year												
Japanese × 2003 (ref)												
Japanese × 2005 (ref)												
Chinese × 2003	1.12	(0.57, 2.18)	.749	1.19	(0.61, 2.30)	.615	1.18	(0.60, 2.28)	.633	1.11	(0.57, 2.17)	.759
Chinese × 2005	0.75	(0.40, 1.44)	.391	0.75	(0.40, 1.42)	.383	0.74	(0.39, 1.41)	.356	0.65	(0.33, 1.26)	.202
Filipino × 2003	0.89	(0.39, 2.01)	.775	0.85	(0.37, 1.93)	.694	0.83	(0.36, 1.89)	.649	0.85	(0.37, 1.94)	.700
Filipino × 2005	0.91	(0.45, 1.84)	.789	0.88	(0.44, 1.79)	.727	0.88	(0.43, 1.82)	.734	0.85	(0.41, 1.78)	.669
Korean × 2003	0.64	(0.30, 1.39)	.259	0.69	(0.32, 1.48)	.339	0.68	(0.31, 1.46)	.319	0.64	(0.28, 1.46)	.289
Korean × 2005	0.33	(0.15, 0.69)	.**004**	0.32	(0.15, 0.67)	**.003**	0.32	(0.15, 0.68)	**.003**	0.26	(0.12, 0.58)	**.001**
Vietnamese × 2003	1.29	(0.61, 2.76)	.502	1.33	(0.63, 2.80)	.458	1.33	(0.63, 2.81)	.450	1.42	(0.66, 3.05)	.372
Vietnamese × 2005	0.96	(0.48, 1.93)	.906	0.86	(0.43, 1.72)	.677	0.87	(0.43, 1.76)	.708	0.83	(0.41, 1.69)	.605

Age	1.05	(1.03, 1.06)	**<.001**	1.05	(1.03, 1.06)	**<.001**	1.05	(1.03, 1.06)	**<.001**	1.04	(1.02, 1.05)	**<.001**

Age × age	.998	(.997, .999)	**.001**	.998	(.998, .999)	**.003**	.998	(.997, .999)	**.002**	.998	(.998, .999)	**.001**

Gender												
Male (ref)												
Female	1.06	(0.90, 1.25)	.501	1.13	(0.94, 1.37)	.187	1.12	(0.93, 1.35)	.239	1.05	(0.86, 1.27)	.637

Education												
≤ 12 years (ref)												
> 12 years				1.12	(0.90, 1.39)	.308	1.06	(0.85, 1.32)	.593	1.04	(0.83, 1.30)	.721

Marital status												
Not married (ref)												
Married				1.29	(1.04, 1.58)	**.018**	1.28	(1.04, 1.58)	**.019**	1.25	(1.00, 1.56)	**.046**

Employment status												
Not employed (ref)												
Employed				0.67	(0.53, 0.84)	**.001**	0.66	(0.53, 0.83)	**.001**	0.66	(0.52, 0.83)	**<.001**

Federal poverty level												
0-99% (ref)												
100-199%				1.30	(0.98, 1.72)	.074	1.29	(0.97, 1.72)	.076	1.29	(0.96, 1.72)	.089
200-299%				1.42	(1.00, 2.03)	.051	1.36	(0.96, 1.94)	.087	1.32	(0.92, 1.89)	.129
≥ 300%				2.12	(1.53, 2.94)	**<.001**	1.89	(1.37, 2.60)	**<.001**	1.67	(1.20, 2.33)	**.003**

English Proficiency												
Well/not/not at all well (ref)												
NA/very well							1.65	(1.28, 2.11)	**<.001**	1.59	(1.22, 2.06)	**.001**

Health insurance												
No (ref)												
Yes										1.48	(1.09, 2.00)	**.011**

Usual source of care												
No (ref)												
Yes										2.15	(1.49, 3.10)	**<.001**

Most recent doctor's visit												
> 1 year (ref)												
≤ 1 year										2.92	(2.11, 4.05)	**<.001**

P-value, joint significance of added covariates (adjusted Wald test)				**<.001**			**<.001**			**<.001**		

After controlling for differences in education, marital status, employment status and federal poverty level (Model 2), Korean Americans were the only group that had significantly lower screening prevalence than Japanese Americans in 2001, and whose trend to 2005 remained significantly depressed. In addition, being married and having a higher income level (≥ 300% poverty level) were associated with increased odds while being employed was associated with decreased odds of ever receiving CRC screening. Model 3 added English proficiency and Model 4 added health care access as covariates. After adding these covariates, all of which were significantly associated with receipt of CRC screening in the expected direction (higher odds of screening with higher English proficiency and health care access), screening prevalences in 2001 were no longer significantly different among the Asian subgroups. However, the trend in screening prevalence among Korean Americans from 2001 to 2005 remained significantly depressed compared to Japanese Americans. The odds ratio for difference in trend for Korean Americans was fairly stable across all models, ranging from 0.26 to 0.33, suggesting that the added covariates did little to explain the declining trend.

Table 3 presents reasons for not receiving CRC screening reported by respondents who were not up to date with screening. The most common reasons in all Asian subgroups were being unaware of the test and having no health problem. Lack of fit statistics suggested that Korean and Vietnamese Americans had higher than expected rates of citing no health problem as the main reason for not receiving an endoscopy. Vietnamese Americans also had higher than expected rates of citing having no health problems as the main reason for not receiving FOBT, while Japanese Americans had low rates of citing this reason.

**Table 3:** Reasons for not receiving colorectal cancer screening reported by respondents not up to date with screening (past 10 years for endoscopy, past 12 months for FOBT)

	Japanese		Chinese		Filipino		Korean		Vietnamese		Total	
						
	n	%	n	%	n	%	n	%	n	%	n	%
**Reasons for not receiving endoscopy**												
Unaware of test	61	51%	150	53%	110	53%	81	46%	101	47%	503	50%
Have no health problems	23	19%	60	21%	45	22%	65	(**+**)37%	84	(**+**)39%	277	28%
Fear of pain/embarrassed	11	9%	25	9%	15	9%	6	3%	5	2%	62	6%
Other	24	20%	46	16%	39	16%	23	13%	24	11%	156	16%
Total	119	100%	281	100%	209	100%	175	100%	214	100%	998	100%
χ^2^(12) = 46.6, P < .001												

**Reasons for not receiving FOBT**												
Unaware of test	113	68%	213	65%	147	64%	132	59%	126	(-)49%	731	61%
Have no health problems	14	(-)8%	74	22%	48	21%	71	32%	95	(**+**)37%	302	25%
Other	38	(**+**)23%	43	13%	36	16%	20	9%	37	14%	174	14%
Total	165	100%	330	100%	231	100%	223	100%	258	100%	1207	100%
χ^2^(8) = 61.7, P < .001												

Analyses were conducted using chi-square tests of homogeneity.

(+) Cell counts higher than expected under null hypothesis of homogeneity.

(-) Cell counts lower than expected under null hypothesis of homogeneity.

Table 4 provides rates of doctor recommendation among the five Asian subgroups in 2001 and 2005. In 2001, a minority of respondents in all Asian subgroups who were not up to date with screening reported receiving a doctor's recommendation to get screened for CRC in the past year, ranging from 16% among Vietnamese to 31% among Japanese. In 2005, this proportion increased by 16 percentage points among Filipino (p =.009); other groups had statistically insignificant changes. In 2005, only 11% among Korean Americans reported a doctor's recommendation, significantly lower than other groups. Rates among Vietnamese (22%) and Chinese (26%) were also low, while rates among Japanese (33%) and Filipino (39%) Americans were among the highest.

**Table 4:** Proportion of respondents not up to date with colorectal cancer screening (past 10 years for endoscopy, past 12 months for FOBT) reporting doctor recommendation for screening in past year

	2001			2005			Change, 2001 to 2005	
	Proportion	n	Comparisons with p < .05	Proportion	n	Comparisons with p < .05	Proportion	p, difference, 2001 to 2005
**Japanese**	0.31	19/62		0.33	27/83	J/K (p < .001)	0.02	0.809
**Chinese**	0.22	30/138		0.26	67/255	F/C (p=.021)	0.04	0.32
**Filipino**	0.23	25/111	J/V (p=.032)	0.39	44/114	C/K (p < .001)	0.16	0.009
**Korean**	0.18	21/120		0.11	20/180	F/K (p < .001)	-0.07	0.115
**Vietnamese**	0.16	23/141		0.22	26/118	F/V (p=.006)	0.06	0.242

Analyses were conducted using two-sample tests of differences of proportions.

## Discussion

Our analysis shows that differences in screening prevalences between Japanese Americans and Chinese, Filipino, Korean and Vietnamese Americans in California in 2001 were no longer significant after controlling for demographic characteristics and English proficiency. However, the depressed trend in screening from 2001 to 2005 among Korean Americans compared to Japanese Americans was not explained by differences in these variables nor by differences in access to care. Korean Americans are among the more recent immigrant groups and their numbers have increased rapidly since 1965 [14]. They differ from the other Asian ethnic groups studied here in that many are small business owners with no health insurance and have a lower median household income than any other group [15]. Yet our analyses showed that differences in these correlates did not fully explain the decline. Furthermore, Korean Americans were the only subgroup that had a significantly depressed trend.

In addition to demographic and socio-economic characteristics and access to health care, health beliefs influence health care utilization [12]. Thus, the stated reasons for non-adherence with CRC screening guidelines may shed some light on the reasons for this decline. First, about one-third of Korean American respondents stated that they did not get screened because they had no health problem. This suggests that Korean (and Vietnamese) Americans are less familiar with the concept of routine screening to detect health problems before the onset of symptoms than other groups that were included in this analysis. Future programs to promote CRC screening in these Asian subgroups should stress the need for screening *before *symptoms develop. Second, while the proportion of respondents who reported receiving a doctor's recommendation for CRC screening increased from 2001 to 2005 among Filipino Americans and remained stable among Chinese, Japanese and Vietnamese Americans, the proportion trended towards a decrease among Korean Americans. Many studies have shown that a doctor's recommendation is one of the strongest predictors of screening among Asian Americans and other racial/ethnic groups [16-18]. Low levels of health insurance among Korean Americans [19] may explain why they are less likely to receive a doctor's recommendation for screening than other ethnic groups. In addition, Korean Americans who are not comfortable communicating in English may choose a Korean-speaking physician who may be reluctant to recommend CRC screening because of their own lack of knowledge of screening guidelines, their patients' unfamiliarity with the concept of screening and the insufficient health care system referral networks and reimbursement for screening [19]. Finally, a few studies have shown that Korean Americans have other barriers to CRC screening that have not been assessed in CHIS, including lack of transportation and cultural barriers, such as fear of being a burden to one's family if diagnosed with cancer [16]. However, the influence of general and culturally specific barriers on CRC screening among Korean Americans and other Asian populations is poorly understood.

Interestingly, when asked the main reason for non-adherence to CRC screening, very few respondents in these Asian subgroups cited cost, lack of insurance or lack of a doctor. Even after consolidating these reasons into a single category reflective of access to care, the cell counts were too low to allow us to evaluate this as a separate category in the analysis. Hence we collapsed these reasons into the "Other" category in Table 3. Instead, the most frequently cited reason for non-adherence to CRC screening was "being unaware of the test".

Correlates of CRC screening that we found in this large California population-based sample of Asian Americans are similar to those found among African Americans, Latinos, non-Hispanic whites and in other Asian American samples [8,10,18,20,21]. Odds of CRC screening increased with being married, having a higher level of income, having a higher level of acculturation, having health insurance, a usual source of care and a doctor's visit within the past year. In our sample, highest odds ratios were associated with recent doctor's visit, having a usual source of care and high acculturation. Only few studies have examined the association between employment and screening and employment has not emerged as an important correlate in the screening literature [10,18,20,21]. Our finding that unemployed Korean Americans are more likely to be screened for CRC is unexpected. However, it is consistent with two prior studies among Korean Americans [8,16]. In one of our early studies, we found that full- or part-time employment was associated with lower odds of CRC screening among Korean American women, but not among Filipino American women [8]. In a recent study we found that employed Korean Americans residing in Los Angeles were less likely to be screened than those who were unemployed [16]. In that study, 15% of respondents stated "not being able to take time off work" as a reason for not getting CRC screening, which may be one of the reasons for this unexpected correlation. Future studies should further explore this potential barrier to screening.

### Strengths and Limitations

All data are based on self-report which may be inaccurate due to recall and social desirability biases. Some of the tests that were reported may have been diagnostic tests among symptomatic patients or tests that do not qualify as screening tests, such as a single FOBT in a doctor's office. Similarly, self-report of a doctor's recommendation to get screened may be more likely to be recalled by subjects who perceive CRC screening as important than by subjects who don't perceive CRC screening as important. Although the data set has many strengths such as population-based sampling, inclusion of non-English speaking Asian Americans and assessment of some important correlates of screening, it lacked in-depth information on provider recommendation, knowledge of screening guidelines and attitudes regarding CRC screening, all of which have been related to screening utilization in prior studies among Asian Americans [17,22,23].

## Conclusion

Differences in demographic characteristics, English proficiency and access to care explain disparities in 2001 CRC screening prevalence among Japanese, Chinese, Filipino, Korean and Vietnamese Americans residing in California, but even after controlling for these factors, a significant decline in CRC screening between 2001 and 2005 among Korean Americans as compared to Japanese Americans persists and remains unexplained. Future studies that provide a better understanding of the reasons for this decline will be crucial for developing culturally appropriate interventions to promote CRC screening. Our data also suggest a need to more fully understand the role and behavior of providers that serve the Korean community in recommending CRC screening to their Korean American patients.

## Competing interests

The authors declare that they have no competing interests.

## Authors' contributions

AEM and CMC conceptualized the manuscript, AEM led the writing. CMC conducted the analyses and substantially contributed to the manuscript. CMA and PL conducted data management and assisted with data analysis. All authors read and approved the final manuscript.

## Pre-publication history

The pre-publication history for this paper can be accessed here:

http://www.biomedcentral.com/1471-2407/10/214/prepub
